# Down-regulation of IFITM1 and its growth inhibitory role in cervical squamous cell carcinoma

**DOI:** 10.1186/s12935-017-0456-0

**Published:** 2017-10-10

**Authors:** Weinan Zheng, Zhimin Zhao, Xinan Yi, Qiangqiang Zuo, Hongtao Li, Xiaoqing Guo, Dongmei Li, Hongchang He, Zemin Pan, Peiwen Fan, Feng Li, Yanhong Liao, Renfu Shao

**Affiliations:** 1 Department of Biochemistry and Molecular Biology, Department of Human Anatomy and Histology and Embryology, Basic Medical Science of Tongji Medical College, Huazhong University of Science and Technology, Wuhan, 430030 Hubei China; 2 Department of Biochemistry and Molecular Biology, School of Medicine, Shihezi University, Xinjiang Endemic and Ethnic Disease and Education Ministry Key Laboratory, Shihezi, 832002 Xinjiang China; 3 Department of Human Anatomy, Basic Medical Science Division, Hainan Medical University, Haikou, 571100 Hainan China; 4 Genecology Research Centre, Centre for Animal Health Innovation, Faculty of Science, Health, Education and Engineering, University of the Sunshine Coast, Maroochydore, DC, QLD 4558 Australia; 5 Department of Biochemistry and Molecular Biology, School of Medicine, Shihezi University, Shihezi, China

**Keywords:** *IFITM1* gene, Cell proliferation, Migration and invasion, Cervical squamous cell carcinoma

## Abstract

**Background:**

Cervical cancer is a major cause of death in women worldwide. Interferon-induced transmembrane protein 1 (IFITM1) is involved in antivirus defense, cell adhesion, and carcinogenesis in different tissues. However, the role of *IFITM1* gene in cervical squamous cell cancer is unclear.

**Methods:**

To explore the role of IFITM1 in carcinogenesis of cervical cancer, we investigated the expression of *IFITM1* gene in cervical squamous cell carcinoma. *IFITM1* mRNA level was measured by real-time quantitative RT-PCR in cervical cancer tissues and their adjacent normal tissues. IFITM1 protein level was measured by immunohistochemistry. Methylation in the *IFITM1* gene promoter was detected by methylation-specific PCR. We then transfected HeLa cells with *IFITM1* expression vector or control vector. IFITM1 expression was examined; cell migration and invasion were analyzed by wound healing assay and matrigel-coated transwell migration assays, respectively. HeLa cell proliferation was measured by cell counting kit-8 assay and cell cycle analysis. Cell apoptosis was analyzed by Annexin V/propidium iodide double staining assay.

**Results:**

The difference in IFITM1 protein expression between samples from chronic cervicitis and cervical carcinoma was statistically significant (*P* < 0.01). Ki-67 and PCNA protein expression levels were significantly higher in cervical cancer tissues than in their corresponding cervicitis tissues (*P* < 0.05 and *P* < 0.001, respectively). *IFITM1* mRNA level was significantly lower in cervical cancer tissues than in normal cervical tissues (*P* < *0.05*). Methylation of the *IFITM1* gene promoter was significantly higher in cervical cancer than in normal cervical tissues (*P* < 0.05). Transfection of the *IFITM1* pcDNA3.1 construct decreased cell migration and invasion of HeLa cells, inhibited cell proliferation, and increased cell apoptosis.

**Conclusion:**

*IFITM1* gene expression may reduce the proliferation, migration, and invasion of cervical squamous cancer cells.

## Background

Cervical cancer is a major cause of death in women worldwide, with approximately 500,000 new cases and 280,000 deaths reported each year [1]. In China, 75,000 new cases are diagnosed every year; 35% of these patients have recurrent diseases. Multidrug resistance and resistance to radiotherapy are the main causes of recurrent cervical cancer cases, in which conventional treatment methods are ineffective [2]. Although much progress has been made in cervical cancer research, reliable biomarkers to predict the development of cervical cancer tumors are still lacking [3].

Developed technologies, such as gene expression analysis, can be used to identify genetic alterations related to the development of cervical cancer; such alterations are potential biomarkers for the diagnosis and prognosis of cervical cancer patients [4–6]. Previous studies demonstrated the overexpression of the DeltaNp63alpha gene together with p53 gene inactivation in squamous cell cancer (SCC) and down-regulation of the expression of the *C/EBPα* gene in cervical SCC [7, 8]. Overexpression of the *Beclin1* gene in CaSki cells may enhance apoptosis signaling induced by anticancer drugs [9]. Moreover, epigenetic modifications are involved in cervical tumorigenesis; for example, methylation of CpGs, especially in the promoter region of genes, has been suggested as a possible factor influencing the activity of cervical cancer-related genes [10, 11].

We compared the gene expression profiles between cervical cancer tissues and their corresponding normal cervical tissues in our previous study [12]. We found that the mRNA expression level of the interferon-induced transmembrane gene (*IFITM1*) is reduced in cancer tissues [12]. The IFITM protein is a superfamily with a transmembrane domain and an inserting conserved intracellular loop [13]. Over 30 superfamily members of IFITM are involved in antivirus defense, immune cell signaling transduction, cell adhesion, carcinogenesis, and germ cell maturation [14].

IFITM1 is involved in several types of cancers, e.g. it promotes the aggressiveness of colorectal cancer cells [15]. Over expression of IFITM1 enhances the aggressive phenotype of triple-negative SUM149 of inflammatory breast cancer cells [16]. However, over expression of IFITM1 negatively regulated cell growth, whereas suppression of IFITM1 blocked the antiproliferative effect of IFN-gamma, accelerated the cell growth rate and conferred tumorigenicity to a non-malignant hepatocyte in nude mice [17]. In addition, higher IFITM1 expression correlated with improved survival in chronic myeloid leukemia patients [18]. The present study focused on the effect of *IFITM1* gene on the carcinogenesis and development of cervical cancer.

## Methods

### Tissue samples

Between 2008 and 2014, clinical data and cervical SCC samples from patients were collected at the First Affiliated Hospital and the Third Affiliated Hospital of the Medical College of Shihezi University in Xinjiang, China, with the approval of the ethical committee of each hospital. Written informed consent was obtained from patients. Patients received neither chemotherapy nor radiotherapy before sample collection. The diagnoses were confirmed independently by two experienced pathologists. Cervical SCC tissues and adjacent normal cervical tissues were collected from each patient. Tissue samples were frozen immediately after removal and stored at − 80 °C.

### Immunohistochemical staining

Tumor tissues were fixed in 10% formalin, embedded in paraffin, and cut into 4 μm-thick sections. For immunohistochemical staining, tissue sections were deparaffinized in xylene and rehydrated in descending grades of ethanol. Endogenous peroxidase activity was blocked with methanol containing 0.3% H_2_O_2_ for 30 min and then washed in PBS. Antigen retrieval was performed by microwaving with citrate phosphate buffer (pH 6.0). The sections were then placed with the primary antibodies at 4 °C overnight. After incubation, the sections were washed in PBS for 3 min. The sections were then washed five times with PBS for several seconds, incubated with secondary antibodies at 37 °C for 30 min, and washed twice with PBS. After incubation with the secondary antibodies, staining was completed using anti-mouse peroxidase and DAB substrate. Tissue sections were counterstained with hematoxylin. IFITM1, Ki-67, and PCNA protein signals were scored on the following scale considering both the proportion of cells stained and the intensity of staining in those cells: score 0, no cells stained; score 1, weak or absent nuclear staining and < 5% of cells stained; score 2, nuclear staining and between 5 and 25% of the cells stained; score 3, nuclear staining and between 26 and 50% of the cells stained; and score 4, nuclear staining and more than 50% of the cells stained. Two observers scored independently using this scale.

### Real-time RT-PCR

Total RNA was extracted from cell or tissue samples using TRIzol reagent according to the manufacturer’s protocol (Invitrogen). The RNA concentration was determined by agarose gel electrophoresis or absorbance at 260 nm. cDNA was synthesized with Invitrogen’s SuperScript One-Step RT-PCR Kit; each reaction contained 2 μg of total RNA, 2 μL of Oligo(dT) (500 μg/mL), and 7.5 μL of DEPC water. Reactions were heated for denaturation at 65 °C for 5 min and then quenched on ice for 5 min. The following reagents were added to each reaction: 4 µL of 5 × first buffer, 2 µL of 0.1 M DTT, 1 µL of dNTPs (10 mM each), 0.5 μL of RNase inhibitor (40 U/μL), and 1 μL of M-MLV (200 U/μL); the total volume of each reaction was 20 μL. The reactions were maintained at 25 °C for 10 min and 37 °C for 1 h. The reactions were terminated at 70 °C for 10 min. *IFITM1* mRNA quantitative PCR amplification was performed on Light Cycler 480 (Roche Diagnostics) with the forward primer 5′-ACTCAACACTTCCTTCCCCAA-3′ and the reverse primer 5′-CTTCCTGTCCCTAGACTTCACG-3′. The amplicons were 231 bp in size. To normalize the amount of cDNA in each sample, the housekeeping gene glyceraldehyde-3-phosphate dehydrogenase (GAPDH) was quantified in the control experiment with specific primers (forward: 5′-TGTTGCCATCAATGACCCCTT-3′; reverse: 5′-CTCCACGACGTACTCAGCG-3′); the amplicons were 202 bp in size. PCR reactions were performed in a volume of 20 μL, consisting of 10 μL of 2 × PCR buffer, 500 ng of cDNA, 10 μL of 2 × PCR buffer for EvaGreen, 0.6 μL of 20 × EvaGreen, 0.6 μL of forward primer and reverse primer (10 μM) each, and 0.3 μL of Cap Taq polymerase (5 U/μL). DEPC water was added to bring the volume to 20 μL. Reaction conditions were as follows: initial denaturation for 5 min at 95 °C, followed by 40 cycles of denaturation for 15 s at 95 °C, primer annealing for 15 s at 55 °C, extension for 20 s at 72 °C, and UPL fluorescence measurement for 3 s at 76 °C. The *GAPDH* gene was used as an endogenous control to normalize the difference in the amount of cDNA in each sample.

### DNA preparation and detection of HPV

DNA was extracted with an SK1252 genomic DNA isolation mini kit (Shanghai Sangon Biological Engineering Technology and Services Company) according to the manufacturer’s protocol. HPV detection and typing were performed in all samples; HPV16 and HPV18 were analyzed by PCR. The primers used were pHPV16-F (5′-GACCCAGAAAGTTACCACAG-3′) with pHPV16-R (5′-CACAACGGTTTGTTGTATTG-3′) for HPV16 virus detection, as well as pHPV18-F (5′-TGCCAGAAACCGTTGAATCC-3′) with pHPV18-R (5′-TCTGAGTCGCTTAATTGCTC-3′) for HPV18 virus detection. Both HPV16 and HPV18 PCR amplicons were 268 bp in size.

### DNA modification by bisulfite treatment and methylation-specific PCR (MSP)

The DNA samples were subjected to bisulfite treatment using CpGenome™ DNA modification kit S7820 (CHEMICON, Temecula, CA, USA) according to the manufacturer’s protocol. The modified DNA was purified, followed by ethanol precipitation and then stored at − 80 °C. Methylated primers were pM-IFITM1-F (5′-GAGATTTTCGTGTTCGATTATGTC-3′) and pM-IFITM1-R (5′-ATAAAACCCCAAACTCACCG-3′), whereas unmethylated primers were pUM-IFITM1-F (5′-AGATTTTTGTGTTTGATTATGTTGT-3′) and pUM-IFITM1-R (5′-ATAAAACCCCAAACTCACCAAC-3′). Each reaction was 25 μL containing 1 μL of modified DNA template, 0.5 μL of forward and reverse primer each (0.5 μmol/L), 12.5 μL of Taq DNA Polymerase Mix, and 10.5 μL of H_2_O. The reaction conditions were as follows: 34 cycles at 95 °C for 5 min, 94 °C for 1 min, 58 °C for 1 min, and 72 °C for 1 min, and an extension step at 72 °C for 10 min. PCR results were checked by agarose gel (2%) electrophoresis.

### RT-PCR

mRNA expression in the 10 methylated and 10 unmethylated cervical tissues was measured by RT-PCR. Total RNA was extracted with TRIzol Reagent (Life Technologies) according to the manufacturer’s instructions. RNA was subjected to cDNA synthesis using an oligo(dt) primer and reverse transcriptase (Fermentas). About 2 μL of cDNA products was then PCR-amplified using *IFITM1* gene primers and Taq DNA Polymerase Mix according to the manufacturer’s instructions. RT-PCR primers were pRT-IFITM1-F (forward: 5′-ATGTCGTCTGGTCCCTGTTC-3′) and pRT-IFITM1-R (reverse: 5′-GTCATGAGGATGCCCAGAAT-3′). *GAPDH* served as the internal control; the primers were GAPDH-F (forward, 5′-GCCAAAAGGGTCATCATCTC-3′) and GAPDH-R (reverse, 5′-GTAGAGGCAGGGATGATGTTC-3′). 5 μL of PCR products was run on 1% agarose gel to determine *IFITM1* gene expression.

### Cell culture and transfection

cDNA of *IFITM1* gene was cloned into pcDNA3.1(−) expression vector and confirmed by gene sequencing. In addition, pcDNA3.1(−) vectors were used as the control. HeLa cells were plated at 2 × 10^5^ cells per well in a six-well cell culture plate at 24 h before transfection. Subsequently, 2 μg of *IFITM1* constructs and the control vector were mixed with 6 μL of Lipofectamine 2000 (Invitrogen). The mixture was incubated at room temperature for 10 min. After washing the cells with 1 × PBS, the DNA/Superfect mixtures were transferred to HeLa cells. The control pcDNA3.1 vector was transferred into HeLa cells separately. Transfected HeLa cells were then incubated at 37 °C in 5% CO_2_ for 24 h. The effect of *IFITM1* overexpression was determined by measuring immunofluorescence luciferase activity using an assay system according to the manufacturer’s protocol (Promega). Each experiment was repeated with multiple batches of cells.

### Wound healing assay

Cells were seeded onto six-well plates (1 × 10^5^ cells/dish). When cells reached over 90% confluence, a scratch was made across the cell monolayer with a tip. Cells were gently washed with PBS three times and maintained in a fresh medium. Cells were incubated for 24 h and photographed using an inverted tissue culture microscope at 100 × magnification. Assays were performed at least three times, and data are presented as the mean ± SD. The migration potential between *IFITM1* expressing cells and control cells was compared by relative gap distance. *P* < 0.05 was considered statistically significant.

### Cell migration and invasion assays

Cells were serum-starved overnight. The top chambers of 6.5 mm Corning Costar transwells (Corning, USA) were loaded with 0.2 mL of cells (5 × 10^5^ cells/mL) in serum-free media. Complete media (0.6 mL) were added to the bottom wells, and cells were incubated at 37 °C overnight. Cells that migrated through the membrane were fixed, stained with 0.1% crystal violet, and examined under a light microscope. Images of the cells at the bottom of the membrane were captured using a Canon camera and a Zeiss microscope. The mean values were obtained from three individual experiments using Student’s *t* tests. For cell invasion assay, cells were serum-starved overnight. The 24-well cell culture inserts (8 mm pore size, BD Biosciences, San Jose, USA) were loaded with 0.5 mL of cells (5 × 10^5^ cells/mL) in serum-free media. Complete media (0.5 mL) were added to the bottom wells, and cells were incubated at 37 °C for 24 h. Cells were fixed, stained, and analyzed as described above.

### Cell proliferation determined by cell counting kit-8 assay

Proliferation of HeLa cells transfected by the *IFITM1* pcDNA3.1 construct and pcDNA3.1 was determined using cell counting kit-8 reagents (Dojindo Laboratories, Japan). HeLa cells were seeded in 96-well plates (Falcon; Becton–Dickinson Labware) at 2 × 10^5^ cells per well in DMEM containing 10% FBS. The cells were incubated for 24, 48, 72 h at 37 °C. Spectrometric absorbance at 450 nm was measured with a microplate reader (X-Mark; Bio-Rad, USA); the quantification was repeated five times.

### Cell cycle analysis

*IFITM1*—pcDNA3.1 construct- or pcDNA3.1 vector-transfected cells were digested by trypsin, fixed with 70% ethanol, and stored at 4 °C until analysis. The cells were suspended in 100 μL of 180 μg/mL RNA enzyme A in and then incubated for 30 min at room temperature. Propidium iodide (PI) solution (50 μg/mL; Merck, Darmstadt, Germany) was added to the cells. The cells were then kept in a dark room for 15 min. The DNA content of cells was measured by flow cytometry (FACScan system, Becton–Dickinson). The G_0_/G_1_ phase ratio, as well as S and G_2_/M phase ratio, was determined from flow cytometry data.

### Cell apoptosis detected by flow cytometry

Apoptosis was analyzed by Annexin V-fluorescein isothiocyanate staining using an Alexa Fluor 488 Annexin V kit (Invitrogen). In total, 1.0 × 10^6^ of *IFITM1*—pcDNA3.1 construct-transfected cells, as well as the same amount of pcDNA3.1 vector-transfected cells, were washed twice with cold PBS. Cells were then resuspended in 1 × Annexin-binding buffer for 30 min. Cell apoptosis was determined by Annexin V-fluorescein isothiocyanate/PI double staining. Cell transfection was analyzed on FACS calibration with the software Cell Quest (Becton–Dickinson). All experiments were carried out in triplicate.

### Statistical analysis

SPSS17 software was used in all statistical analyses. IFITM1 protein levels were compared between cervical carcinoma and chronic cervicitis using non-parametric test. Results of the triplicates were represented as the mean ± standard deviation (if applicable). Results were considered statistically significant if *P* < 0.05 in two-tailed Student’s *t* tests.

## Results

### IFITM1, Ki-67, and PCNA protein expression in cervical cancer and chronic cervicitis tissues measured by immunohistochemistry

**Fig. 1:**
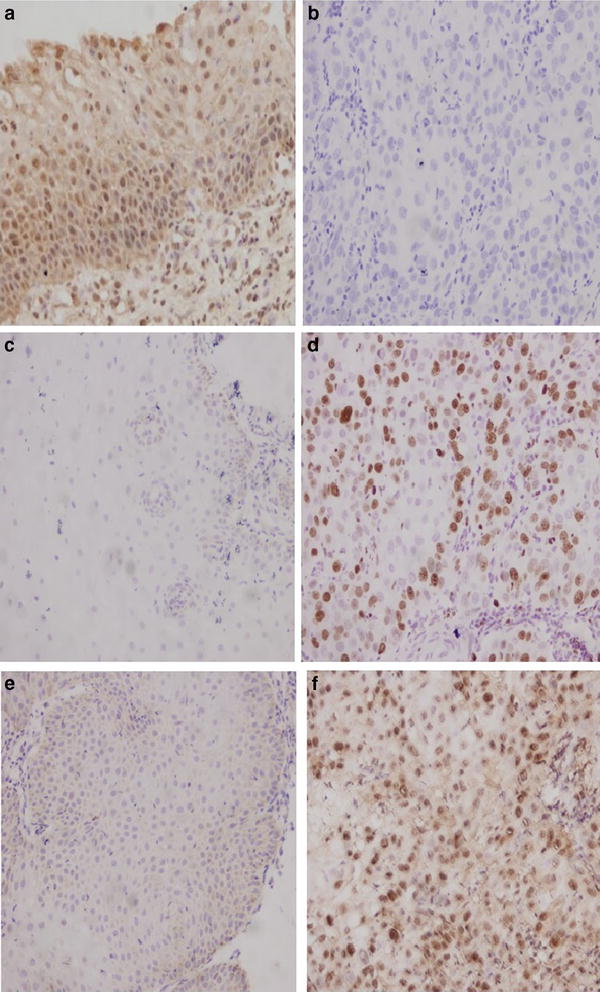
IFITM1, Ki-67, and PCNA protein expression level in cervical cancer tissues and cervicitis tissues. **a** IFITM1 protein expression in chronic cervicitis tissues (× 200); **b** IFITM1 protein expression in cervical cancer tissues (× 200); **c** Ki-67 protein expression in chronic cervicitis tissues (× 200); **d** Ki-67 protein expression in cervical cancer tissues (× 200); **e** PCNA protein expression in chronic cervicitis tissues (× 200); **f** PCNA protein expression in cervical cancer tissues (× 200)

A total of 55 cervical cancer and 40 chronic cervicitis paraffin tissues were cut into triplicate sections to analyze the expression of IFITM1, Ki-67, and PCNA proteins. IFITM1 protein was stained brown in the cell membrane and cytosol of cervical tissue cells by immunohistochemistry (Fig. 1a, b). Ki-67 and PCNA protein expression was stained brown in the nucleus of cervical tissue cells by immunohistochemistry (Fig. 1c–f). IFITM1 protein expression in chronic cervicitis tissues was higher than that in cervical cancer tissues. However, in the corresponding chronic cervicitis tissues, the protein expression levels of Ki-67 and PCNA were both lower than those in the corresponding cervical cancer tissues.

IFITM1 protein expression significantly decreased in cervical cancer tissues than in chronic cervicitis tissues (*P* < 0.01; Table 1). By contrast, Ki-67 protein level significantly increased in cervical cancer tissues than in chronic cervicitis tissues (*P* < 0.01; Table 1). In addition, PCNA protein level was significantly higher in cervical cancer tissues than in the corresponding chronic cervicitis tissues (*P* < 0.001; Table 1).

**Table 1:** Expression level of IFITM1, Ki-67, and PCNA proteins in cervical cancer and chronic cervicitis tissues

Group	IFITM1				Ki-67				PCNA			
	Score 4	Score 3	Score 2	Score 0–1	Score 4	Score 3	Score 2	Score 0–1	Score 4	Score 3	Score 2	Score 0–1
Chronic cervicitis tissues	5	18	11	6	7	4	9	20	2	12	9	17
Cervical cancer tissues	2	12	19	22	27	6	4	18	22	25	6	2
P value		< 0.01					< 0.01			< 0.001		

### Methylation of the IFITM1 gene promoter in cervical cancer and normal cervical tissues

**Fig. 2:**
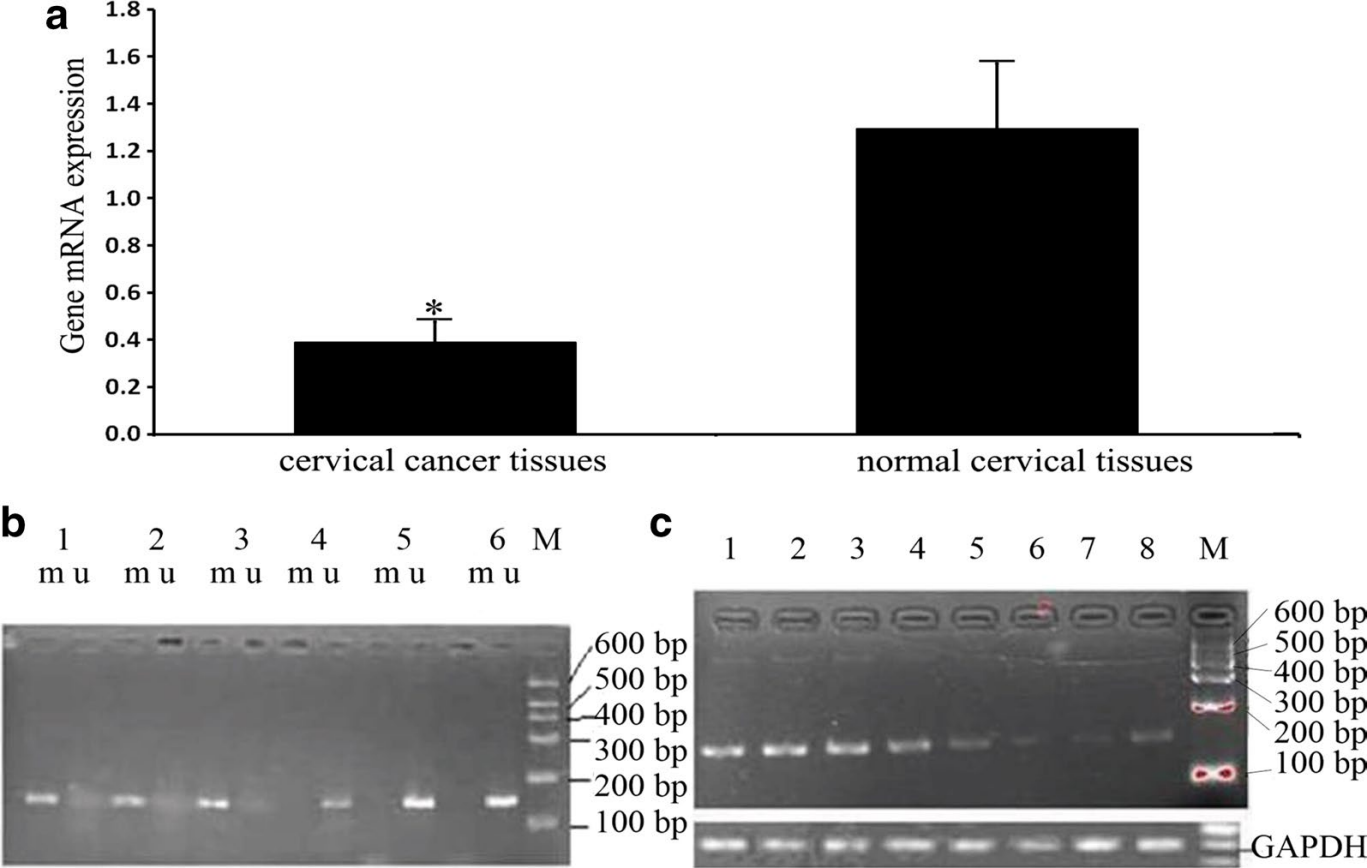
IFITM1 gene methylation analysis and mRNA expression analysis. **a** *IFITM1* mRNA expression level in 24 cervical cancer tissues significantly decreased than that in 15 adjacent normal cervical tissues by real-time quantitative RT-PCR (*P* < 0.05). **b** Methylation of the *IFIMT1* gene promoter was analyzed by MSP: 1–3 cervical cancer tissues; 4–6 normal cervical tissues; M: Marker (100–600 bp); m: production of methylation via MSP; u: production of non-methylation via MSP. **c** *IFITM1* mRNA expression in the *IFITM1* gene promoter of methylated and unmethylated cervical tissues: 1–4 the *IFITM1* gene in unmethylated normal cervical tissues; 5–8 the *IFITM1* gene promoter in methylated samples; M: Marker (100–600 bp); GAPDH as an internal control

*IFITM1* mRNA expression in 24 cervical cancer tissues was 0.39 ± 0.088, which was significantly lower than that in 15 adjacent normal cervical tissues at 1.295 ± 0.276 (t = − 2.401, *P* < 0.05) (Fig. 2a). Methylation analysis of the *IFITM1* gene promoter by MSP revealed 47 samples of *IFITM1* gene methylation in 60 cervical cancer tissues and five samples of *IFITM1* gene methylation in 60 normal cervical tissues (Fig. 2b); the difference was statistically significant (*P* < 0.001; Table 2). HPV16 and HPV18 infections in cervical cancer and normal cervical tissues were detected by PCR. A total of 44 HPV16 and HPV18 infections were found in 60 cervical cancer tissues. By contrast, seven HPV16 and HPV18 infections were noted in 60 normal cervical tissues; the difference was statistically significant (*P* < 0.05; Table 2). The *IFITM1* gene methylation rate in 44 HPV16 and HPV18 infection-positive samples was higher than that in 16 HPV16 and HPV18 infection-negative samples; the difference was statistically significant (*P* < 0.05; Table 3).

**Table 2:** Methylation in the *IFITM1* gene promoter and infection of HPV16 and HPV18 in cervical cancer and normal cervical tissues

Group	Number	Methylation	HPV16 and HPV18 infection	Methylation rate (%)	HPV infection rate (%)
Normal cervical tissues	60	5	7	8.3	11.7
Cervical cancer tissues	60	47	44	78.3	73.3

Mann–Whitney test: IFITM1, *P* < 0.001; χ² test: HPV16 and HPV18, *P* < 0.05

**Table 3:** Methylation in the *IFITM1* gene promoter in HPV16 and HPV18 positive and negative samples

Group	Number	Methylation	Non-methylation	Methylation rate (%)
HPV16 and HPV18 positive	44	38	6	86.36
HPV16 and HPV18 negative	16	9	7	56.25

Fisher’s exact test: *P* < 0.05

In addition, the *IFITM1* gene promoter in methylated cervical cancer tissues at the mRNA expression level was lower than that of the *IFITM1* gene promoter in unmethylated normal cervical tissues (Fig. 2c).

### Effect of the IFITM1 gene on HeLa cell migration

**Fig. 3:**
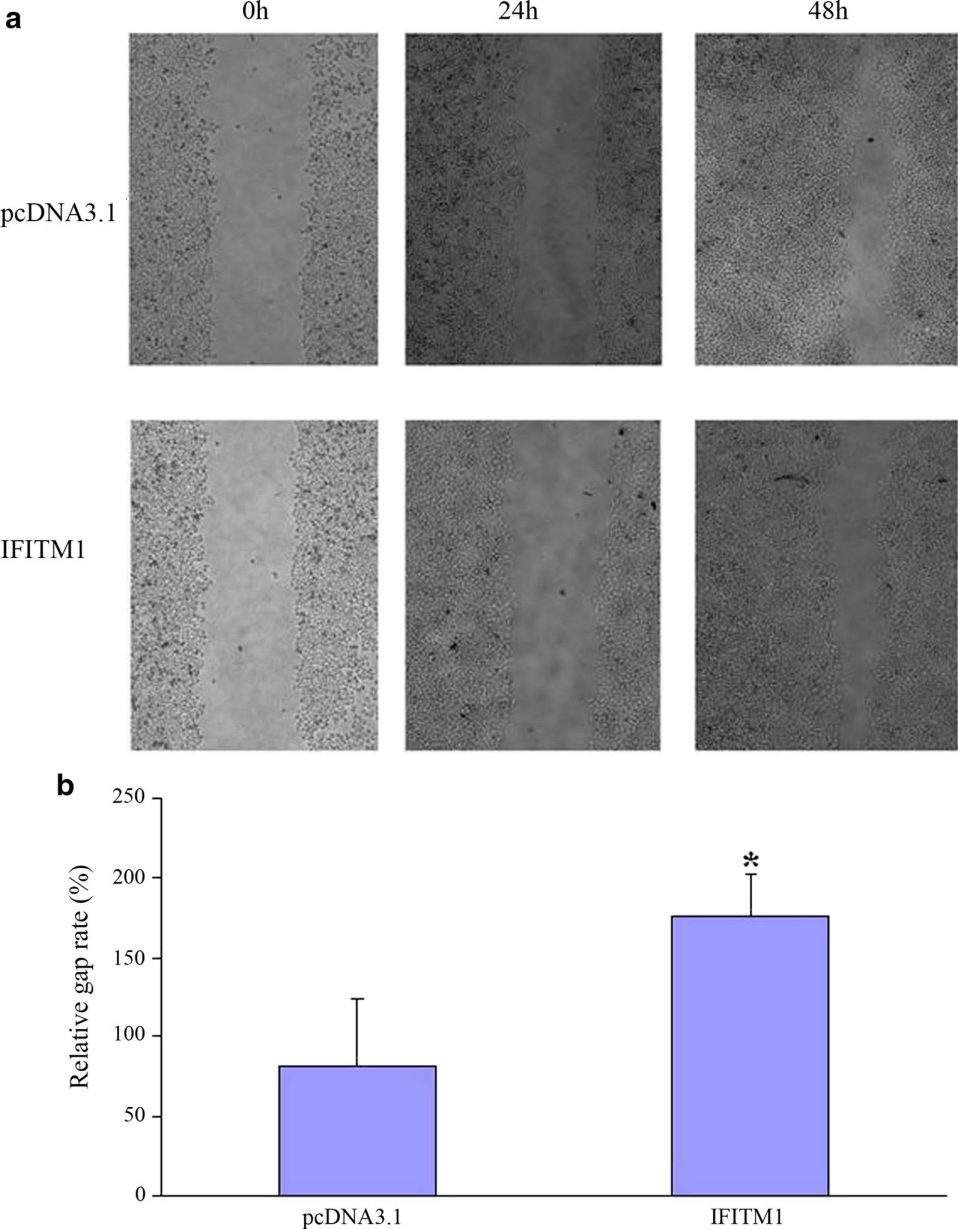
Wound healing assay for analysis of overexpression of the *IFITM1* gene on HeLa cell migration. **a** Overexpression of the *IFITM1* gene in HeLa cells inhibited wound healing capabilities compared with that in pcDNA3.1-transfected HeLa cells. **b** Overexpression of the *IFITM1* gene in HeLa cells inhibited migration compared with that in pcDNA3.1-transfected HeLa cells. The relative gap rate in the *IFITM1* gene-transfected group was significantly greater than that in the pcDNA3.1-transfected group (*n* = 3, *P* < 0.05)

The *IFITM1* gene recombinant construct and pcDNA3.1 vector were transfected into HeLa cells. The gap of wound width was 500 µm, and wound healing was observed at 0, 24, and 48 h. The images taken at these time points showed that the wound width of relative gap rates at 48 h of the *IFITM1* gene recombinant group and pcDNA3.1 vector-transfected group were 175 ± 26.81 and 81 ± 43.25, respectively. The difference was statistically significant (*P* < 0.05). This result indicated that the *IFITM1* gene could inhibit the migration of HeLa cells (Fig. 3).

### Effect of IFITM1 gene on the migration and invasion of HeLa cells by transwell gel assay

**Fig. 4:**
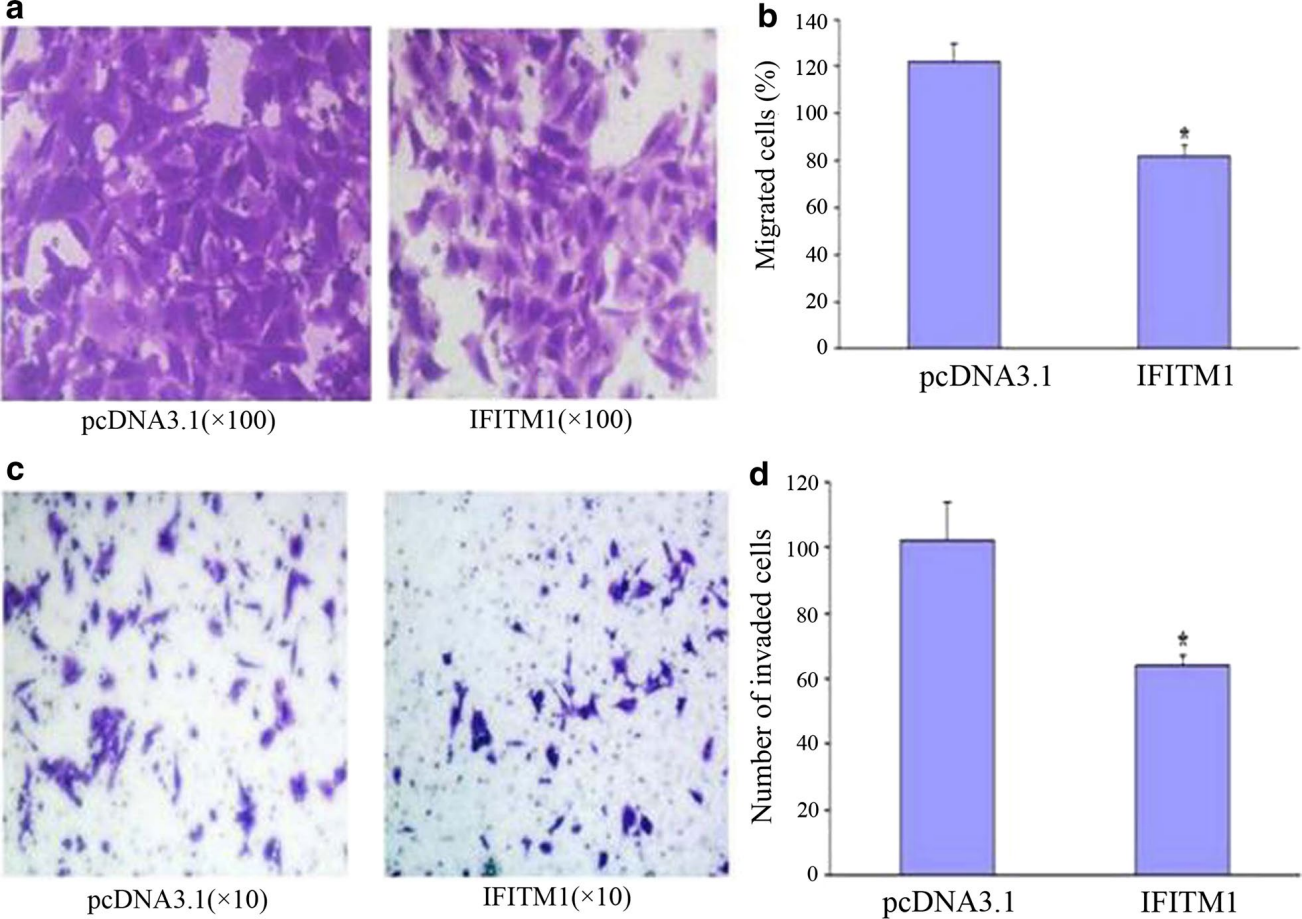
HeLa cell migration and invasion were inhibited by the *IFITM1* gene. **a** HeLa cell migration in the *IFITM1* gene-transfected group was lower than that in the pcDNA3.1 gene-transfected group (× 100). **b** HeLa cell migration in the *IFITM1* recombinant construct was lower than that in the control group; the difference was statistically significant (*P* < 0.05). **c** Invasion of HeLa cells in the *IFITM1* gene-transfected group was lower than that in the pcDNA3.1-transfected group (× 10). **d** HeLa cell invasion in the *IFITM1* gene-transfected group was significantly lower than that in the pcDNA3.1-transfected group; the difference was statistically significant (*P* < 0.05)

Cells of the *IFITM1* gene construct-transfected group and pcDNA3.1 vector-transfected group were seeded in transwells. Cells moved from low nutrition to high nutrition through an 8 µm hole. After 24 h, HeLa cells were stained by crystal violet, and images of the number of HeLa cells were obtained. The numbers of HeLa cells through the micropore in the *IFITM1* gene construct group and pcDAN3.1 control group were 82 ± 4.04 and 122 ± 7.23, respectively. The difference was statistically significant (*P* < 0.05). This finding indicated that overexpression of *IFITM1* gene inhibited the migration of HeLa cells (Fig. 4a).

After transfection by the *IFITM1* gene construct and pcDNA3.1 vector, the invasion of HeLa cells was analyzed by a transwell matrix gel experiment. After 48 h, the number of HeLa cells at the bottom chamber was detected and images were taken. The numbers of HeLa cells of the *IFITM1* recombinant construct group and pcDNA3.1 control group through the micropore were 64.33 ± 2.52 and 102.0 ± 11.53, respectively. The difference was statistically significant (*P* < 0.05). This result showed that overexpression of the *IFITM1* gene inhibited the invasion of HeLa cells (Fig. 4b).

### HeLa cell proliferation measured by cell counting kit-8 assay and cell cycle analysis by flow cytometry

**Fig. 5:**
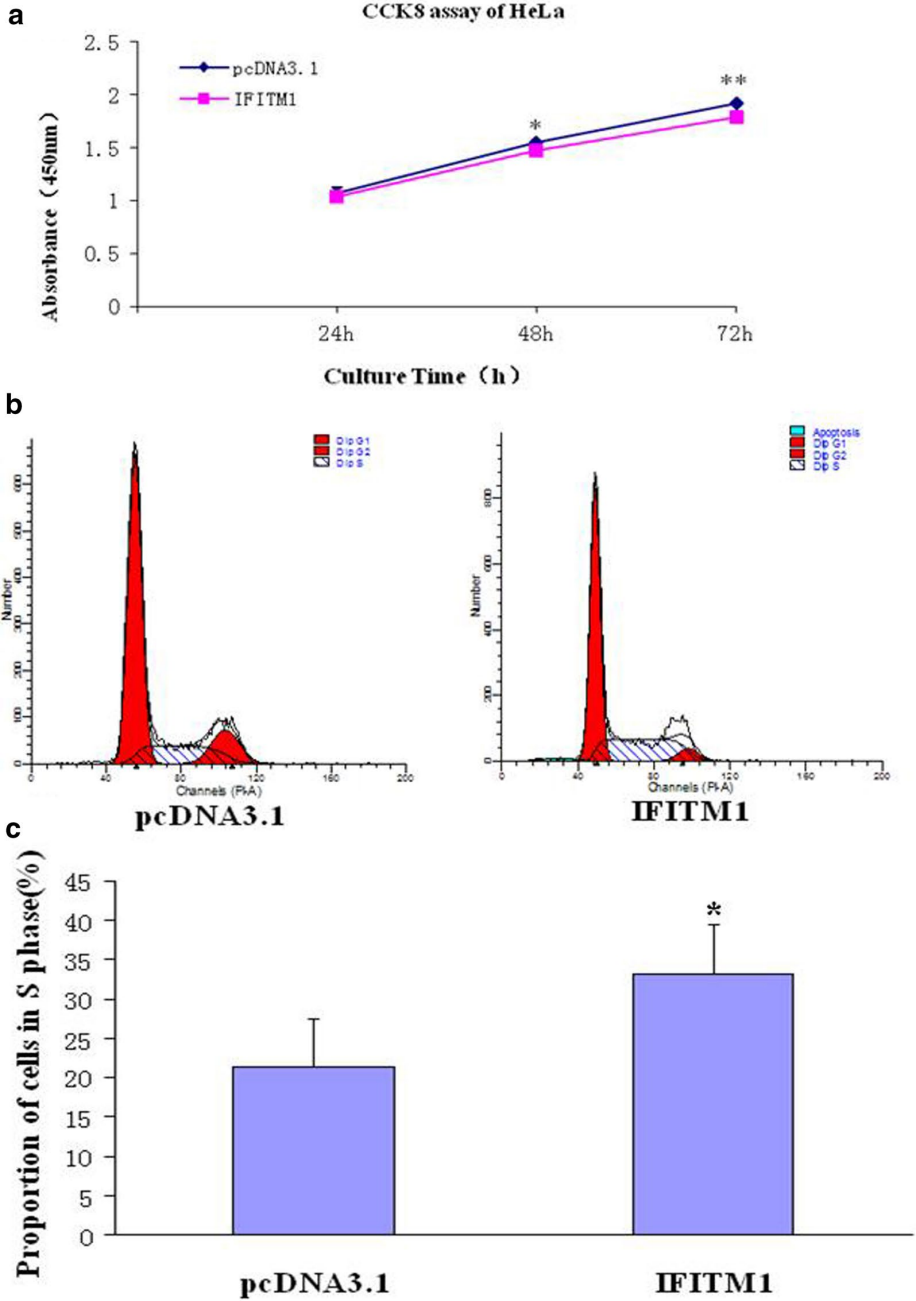
HeLa cell proliferation in the S phase of the cell cycle was inhibited by the *IFITM1* gene. **a** HeLa cell proliferation in the *IFITM1* gene-transfected group was significantly lower than that in the pcDNA 3.1 plasmid control group by cell counting kit-8 assay at 48 and 72 h; the difference was statistically significant (*P* < 0.05). **b** HeLa cell number of the S phase of the cell cycle in the *IFITM1* gene recombinant construct group was greater than that in the pcDNA3.1 control group, as revealed by flow cytometry. **c** HeLa cell number in the S phase of the cell cycle in the *IFITM1* gene-transfected group was significantly greater than that in the pcDNA3.1-transfected group; the difference was statistically significant (*P* < 0.05)

After transfection by the *IFITM1* gene construct and pcDNA 3.1 control vector, HeLa cell proliferation was measured by cell counting kit-8 assay. In terms of cell proliferation, the *IFITM1* construct-transfected group was significantly lower than the pcDNA 3.1 vector control group at 48 and 72 h (*P* < 0.5, *n* = 5) (Fig. 5a). The effect of *IFITM1* gene on the HeLa cell cycle was determined by PI staining, whereas FACS analysis was conducted to detect the DNA contents of cells. The number of cells in the G_1_/G_0_ phase, S phase, and G_2_/M phase can be used to determine the effect of *IFITM1* gene on the cell cycle. After overexpression of *IFITM1* gene, the number of HeLa cells obviously increased in the S phase of the cell cycle. Therefore, *IFITM1* gene overexpression blocked HeLa cells in the S phase. The numbers of HeLa cells in the S phase of the *IFITM1* construct-transfected group and pcDNA3.1-transfected group were 33.21% ± 6.22% and 21.38% ± 6.19%, respectively. The difference was statistically significant (*P* < 0.05; Fig. 5b, c). The *IFITM1* construct-transfected group and pcDNA3.1 vector-transfected group showed no obvious difference in the G_1_ and G_2_/M phase of the HeLa cell cycle.

### Effect of IFITM1 gene on HeLa cell apoptosis measured by Annexin V-FITC-PI assay

**Fig. 6:**
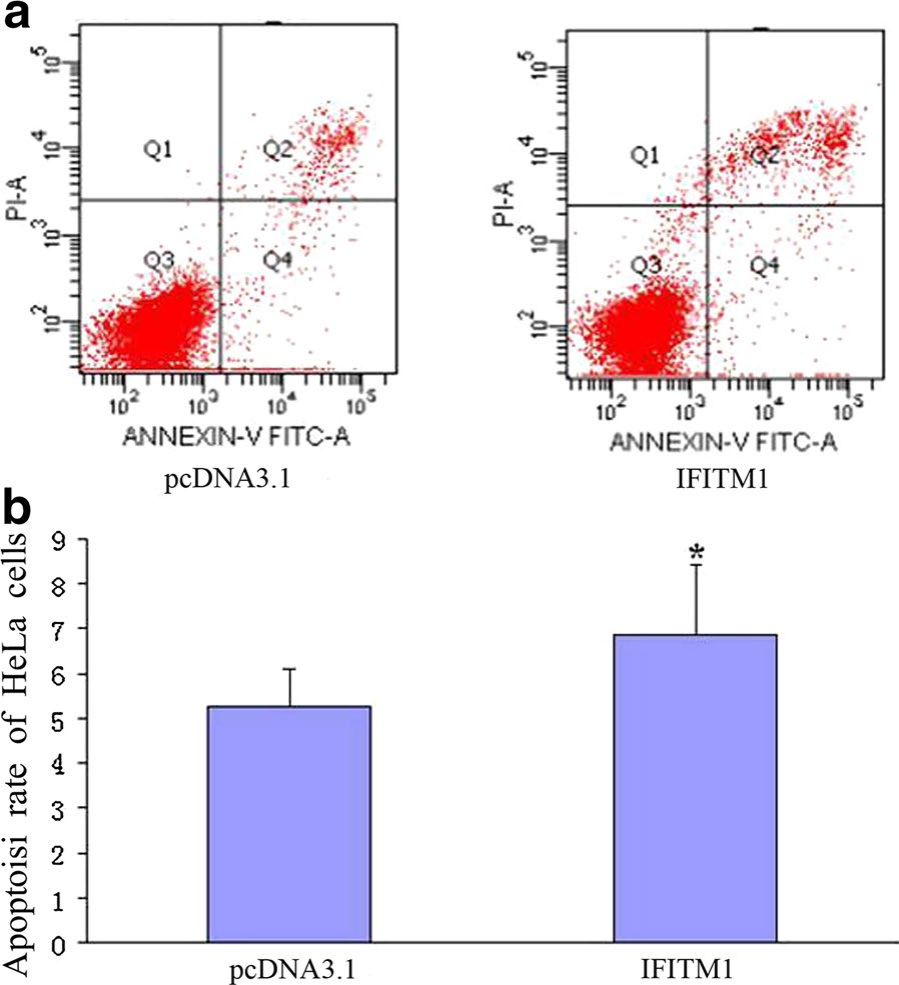
Effect of the *IFITM1* gene on the HeLa cell apoptosis rate analyzed by Annexin V-FITC-PI assay. **a** The apoptosis rates of HeLa cells of the *IFITM1* gene-transfected group and pcDNA3.1-transfected group were quantified by Annexin V/PI staining via flow cytometry. Results shown are the representative examples of flow cytometry. **b** The apoptosis rate of the *IFITM1* gene-transfected group was obviously greater than that of the pcDNA3.1-transfected group; the difference was statistically significant (*n* = 3; *P* < 0.05)

HeLa cell apoptosis was analyzed by Annexin V-FITC-PI assay. The apoptosis rate was 6.88 ± 1.52 in the *IFITM1* construct-transfected group and 5.26 ± 0.83 in the pcDNA3.1 vector-transfected group. Therefore, overexpression of the *IFITM1* gene increased HeLa cell apoptosis (*P* < 0.05; Fig. 6).

## Discussion

*IFITM1* gene is an important factor that controls cell growth, and IFITM1 protein is initially proven to possess Leu-13 protein function. Leu-13 is a known leukocyte antigen, which forms a membrane complex involved in transduction, anti-proliferation, and homotypic adhesion signals in lymphocytes [19]. IFITM1 is an anti-HCV interferon-stimulated gene, and controls HCV infection through the interruption of viral coreceptor function [20]. IFITM1 serves as an important molecule to restrict HCV infection, and it may have implications in the development of therapeutic modalities [21]. IFITM1 restricts an early step in influenza A viral replication. Meanwhile, IFITM proteins confer basal resistance to influenza A virus but are also inducible by types I and II interferons and are critical for an interferon’s virustatic actions [22].

Previous studies found that *IFITM1* gene overexpression can increase cell proliferation, migration, and invasion of esophageal SCC [23], head and neck cancer [24], and glioma [25]. However, *IFITM1* gene expression in cervical cancer tissues was lower than that in normal cervical tissues. The reason why IFITM1 protein expression decreased in cervical cancer tissues is unclear. In the current study, we investigated the expression of IFITM1, Ki-67, and PCNA proteins in cervical cancer tissues and chronic cervicitis tissues. Our results showed that IFITM1 protein significantly decreased in cervical cancer tissues relative to that in chronic cervicitis tissues. However, the expression levels of Ki-67 and PCNA proteins in the corresponding cervical cancer tissues increased relative to those in chronic cervicitis tissues. We found that decreased IFITM1 protein expression resulted in cell proliferation in cervical tissues. Our results were similar to those of a previous study on hepatoma cells, which showed that the overexpression of IFITM1 inhibits cell proliferation [26].

Gene methylation regulates cell growth and is related to carcinogenesis [27–29]. Methylation in the *IFITM1* gene promoter was analyzed in cervical cancer tissues and normal cervical tissues by MSP. Methylation in the *IFITM1* gene promoter in cervical cancer tissues increased significantly compared with that in normal cervical tissues. However, the mRNA expression level in the corresponding cervical cancer tissues decreased. Reduction in *IFITM1* mRNA expression in cervical tissues may lead to cervical carcinogenesis. In addition, we found that *IFITM1* gene inhibited the invasion and migration of HeLa cells, block HeLa cells in the S phase, and enhance apoptosis. However, a previous study showed that IFITM1 plays an important role during invasion at the early stage of head and neck squamous cell carcinoma (HNSCC) progression, and IFITM1 can be a therapeutic target for HNSCC [30]. The reason why IFITM1 showed various effects in different types of cancer remains unknown, and further studies are needed.

## Conclusion

The decrease in IFITM1 protein expression in cervical cancer may lead to cell proliferation. Therefore, the expression level of IFITM1 may be changed to improve the status of cervical cancer patients. In addition, reduced *IFITM1* gene expression in cervical cancer tissues may be a result of methylation of the *IFITM1* gene promoter, which is a potential target for cervical cancer treatment.

## Abbreviations

IFITM1: interferon-induced transmembrane protein 1
SCC: squamous cell cancer
GAPDH: glyceraldehyde-3-phosphate dehydrogenase
PI: propidium iodide
HNSCC: head and neck squamous cell carcinoma
PCR: polymerase chain reaction
HPV: human papillomaviruses
